# Arabic Syntactic Diacritics Restoration Using BERT Models

**DOI:** 10.1155/2022/3214255

**Published:** 2022-10-30

**Authors:** Waleed Nazih, Yasser Hifny

**Affiliations:** ^1^College of Computer Engineering and Sciences, Prince Sattam Bin Abdulaziz University, Al Kharj, Saudi Arabia; ^2^Faculty of Computers and Artificial Intelligence, Helwan University, Cairo, Egypt

## Abstract

The Arabic syntactic diacritics restoration problem is often solved using long short-term memory (LSTM) networks. Handcrafted features are used to augment these LSTM networks or taggers to improve performance. A transformer-based machine learning technique known as bidirectional encoder representations from transformers (BERT) has become the state-of-the-art method for natural language understanding in recent years. In this paper, we present a novel tagger based on BERT models to restore Arabic syntactic diacritics. We formulated the syntactic diacritics restoration as a token sequence classification task similar to named-entity recognition (NER). Using the Arabic TreeBank (ATB) corpus, the developed BERT tagger achieves a 1.36% absolute case-ending error rate (CEER) over other systems.

## 1. Introduction

**Figure 1:**
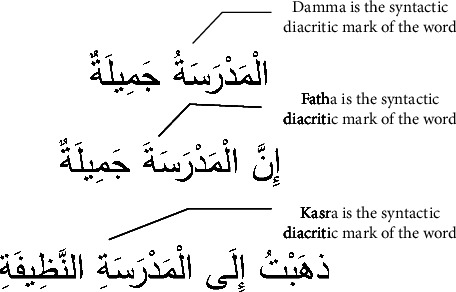
In the given text, the word 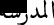 has identical morphological diacritics, but the syntactic diacritics are different.

One of the main problems in Arabic natural language processing is that the Arabic text is often written without diacritics [1, 2]. Words with the same written form have different pronunciations and meanings can be disambiguated with the help of diacritics. Morphological diacritics and syntactic diacritics are the two types of Arabic word diacritics. The morphological diacritics are the diacritics inside a word. Moreover, the syntactic diacritic tag is the last diacritic mark or tag in the stem of a word. The rules of Arabic grammar and the context of a word contribute directly to the prediction of the syntactic diacritic mark. For example, Figure 1 shows three Arabic sentences with diacritics. Based on the Arabic grammar rules, the syntactic diacritics of the word 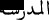 are different. On the other hand, the morphological diacritics are identical.

It was found empirically that diacritization errors are often related to the prediction of the syntactic diacritic task when a machine learning approach is developed [3–5]. Diacritics restoration is a vital component in Arabic text-to-speech (TTS) systems where the phonetic transcription is extremely affected by diacritization errors [6, 7]. When the syntactic diacritics are wrong, the listeners to the output speech are usually disturbed.

Diacritized Arabic text is very essential for many natural language processing (NLP) applications such as speech synthesis and machine translation. Unfortunately, a lot of electronic Arabic resources did not have these diacritics, and this shows the importance of automatic diacritization.

In addition, the restoration of Arabic syntactic diacritics (i.e., the last character of the stem of each word) is more challenging than morphological diacritics. Although most of the state-of-the-art systems have a low error rate in the case of morphological diacritics, they still have a high error rate when restoring syntactic diacritics [3].

Our objective in this paper is to solve this problem based on BERT models and compare our proposed approach with other approaches.

To our knowledge, the proposed model is the first attempt to use BERT models to restore the Arabic diacritics (for other languages, see [8]). Our contributions include the following: (1) formulating syntactic diacritics restoration as a named-entity recognition (NER) problem and using the BERT model for diacritics restoration; (2) Arabic BERT automatic hyperparameters search optimization; (3) using two steps fine-tuned model which used two datasets for model building; and (4) achieving a case-ending error rate (CEER) less than other systems.

The paper is organized as follows: The next section summarizes related work. In Section 3, BERT models or taggers and our problem formulation are reviewed. The data used to train the BERT taggers are described in Section 4.  Section 5 details the experimental work and results on the Arabic TreeBank (ATB) corpus [9]. Finally, the last section presents the summary and our conclusions.

## 2. Related Work

Many approaches have been introduced to address the problem of Arabic diacritics restoration. These approaches can be grouped into rules-based approaches, statistically-based approaches, and deep learning approaches [1]. In the literature, there is plenty of work done for Arabic diacritization, some of it considered milestones such as [5, 10, 11]. In this section, we focused on the most recent work.

Although the earliest work was dependent on predefined rules, this still helps in the diacritization process. Neme and Paumier [12] argued that the supervised machine learning techniques depend on huge annotated datasets which are rare and difficult to be available in the case of the Arabic language.

They suggested utilizing lexical resources and grammar rules to avoid the aforementioned problem. The proposed system (i.e., Arabic-Unitex) replaced each undiacritized word with its equivalent diacritized from the prepared lexicon.

This system was integrated with a set of vowel omission rules. These rules enable the system to restore the diacritized copy of every word from a list of candidates using omission-tolerant dictionary lookup. The prose system is fast in diacritization, but the required lexicon cannot be prepared or updated automatically.

Alansary [13] proposed a rules-based diacritization system called “Alserag.” The proposed system is based on a prepared dictionary containing diacritized words and a set of linguistics rules. Diacritizing dictionary words begins with the Buckwalter analyzer then the AlKhalil engine, followed by a final step to select the best diacritization manually.

In addition, three modules were developed to provide full diacritization (i.e., morphological analyzer, syntactic analysis, and morph-phono-logical processing). Arabic TreeBank [9] was used to evaluate the system. It achieved a lower word error rate compared to the other three statistical systems.

Most of the statistically-based approaches utilized hidden Markov models (HMMs) or *n*-grams to solve the diacritization problem. In HMM, the hidden states represented the words with diacritization while the observations represented the words without diacritization. In addition, a large dataset is always used for estimating the emission and transition probabilities.

Systems that utilized HMM have the problem of out-of-vocabulary (OOV) words that occur when some undiacritized words exist in the test dataset only and do not exist in the training dataset. Khorsheed [14] proposed a character-level diacritization system based on HMMs to handle the OOV problem. The system converts the text character-by-character to its equivalent ASCII codes and injects the characters as a sequence into the HMM. The Viterbi algorithm was used to find the best path through the HMM, which is considered the output diacritics. The system results were comparable to the other state-of-the-art systems.

Darwish et al. [15] proposed a diacritizer of two components; one for diacritizing the word and the second for its case-ending diacritization. The Viterbi algorithm was employed in the first component in addition to stem back-off and morphological patterns. For the second component, ranking based on support vector machine (SVM), morphological patterns, and linguistic rules was used for case-ending (i.e., syntactic) diacritization. The proposed diacritizer achieved a low error rate.

Another alternative to handling diacritization is based on *n*-grams [16]. An *n*-gram is a group of adjacent units where each unit may be a character, a word, or a semi-word. In addition, the language model is always combined with scoring techniques to utilize *n*-grams with high orders [17].

Hifny [18] used byte pair encoding (BPE) to handle the OOV words. The BPE method converts each word to sub-words with different lengths and allows open vocabulary using a dictionary containing sub-words with fixed lengths. The disadvantage of this method was the low performance of syntactic diacritization similar to the HMM-based methods.

Recently, Masmoudi et al. [19] formulated the diacritics restoration of the Tunisian dialect as statistical machine translation (SMT). They considered the text without diacritics as the source language of the SMT, while the target language is the diacritized text. In addition, they prepared a text corpus for the Tunisian dialect. The proposed SMT achieved a high error rate, and so they suggested increasing the corpus size and integrating a rule-based diacritizer with the proposed SMT in the future.

Deep learning is a form of machine learning based on some architectures of neural networks. One of the best advantages of deep learning is the ability to learn data features and extract their hidden relations automatically. For Arabic diacritization, deep learning approaches usually do not require any prior preprocessing or morphological analysis of the dataset [20]. In [21], it was accelerated using hybrid approaches where a morphological and syntactical analyzer is used to assist the neural networks.

The long short-term memory (LSTM) networks can be used for character, word, or sentence classification [22, 23]. Abbad et al. [24] proposed a model composed of an embedding layer that calculates weights for every input character followed by four bidirectional LSTM layers. In addition, a preprocessing step was required to separate the letters and their corresponding diacritics to use the letters as input and their diacritics as output. Furthermore, the proposed model achieved comparable results over a subset of the Tashkeela corpus [25].

Darwish et al. [3] combined a set of character-level features such as stem and part-of-speech (POS) tags with an embedding layer and a bidirectional LSTM. The proposed system restores core word and case ending diacritization. In addition, they tested the system over more than one dataset for standard and classical Arabic. The proposed system achieved a very low error rate compared to other systems.

Deep belief network (DBN) was used in Arabic diacritization for the first time in [26]. The proposed system does not require any preprocessing steps. In addition, they trained the DBN to classify every character with its equivalent diacritized copy. ATB and Tashkeela corpora were used to evaluate the proposed systems. Furthermore, an extra corpus was prepared from the Arabic text of children's stories to measure the error rate of the system.

Restoring diacritization of Arabic poetry has a higher error rate compared to the normal Arabic text, so Abandah et al. [27] used transfer learning for diacritizing Arabic poetry. They tried a two-stage model that uses one classifier for poetry meter classification and the other to predict the diacritics. In addition, three different architectures of the transfer learning model that use the poetry meter classifier were tried. The model with the highest accuracy was composed of two stacks of bidirectional LSTM.

Dealing with the restoration of Arabic diacritics as a tagging problem was introduced in many papers such as [28, 29]. In this case, the tagger generates the diacritics after it has been trained using extracted features from the raw text.

In [29], the bidirectional LSTM was combined with maximum entropy connections between input and output layers which decreases the diacritization error rate. Hifny [4] improved the previous work [29] using a knowledge distillation technique [30]. In addition, a character-level embedding layer was utilized to handle the OOV problem.

Some of the aforementioned work is based on techniques such as LSTM that are slow in training and inference and cannot be parallelized. In addition, there is still room and opportunity to improve results.

## 3. The BERT Tagger

The BERT model is both conceptually simple and empirically powerful. It achieved the best results for many of the eleven tasks in natural language processing [31]. In addition, it is built on the transformer networks introduced by Google in a landmark paper [32]. In this section, we will review the building blocks of the transformer networks and show how to use the BERT tagger for syntactic diacritics restoration.

### 3.1. Self-Attention Networks

The self-attention networks (SANs) are the core idea of the transformer models [32]. These networks learn contextual relations between words or tokens in a text, and they have the ability to capture long-term dependencies. Hence, they replace the recurrent connections used in recurrent neural networks (RNNs). Moreover, they run much faster than RNNs since they run in parallel.

Assuming we have an input matrix and a query vector, in terms of computation, the parts of the input matrix which are similar to the query vector are given attention (i.e., a similarity score between the input matrix and the query vector is computed). After the similarity score is computed, the input matrix is transformed into an output vector. The weighted summation (or average) of the input matrix is the output vector. This leads to a richer representation which is better than the input matrix.

Mathematically, the attention distribution over the input sequence is computed using dot-product attention. The distribution *α*_*tτ*_ is given as follows:

(1) $$\begin{array}{c} \alpha_{t\tau }=\frac{\exp\;\left(\beta x_{t}^{T}W_{q}^{T}W_{k}x_{\tau }\right)}{\sum_{\overset{´}{T}}\exp\;\left(\beta x_{t}^{T}W_{q}^{T}W_{k}X_{\overset{´}{T}}\right)}\text{,} \end{array}$$

where **W**_*q*_, **W**_*k*_ ∈ *R*^*d*_*k*_.*dx*^ are used to transform **x**_*t*_ to the query and key spaces and $\beta =1/\sqrt{d_{x}}$. Conceptually, the self-attention networks compute the attention weights for each token with respect to every other token. The output representation of the self-attention is obtained by the following equation:

(2) $$\begin{array}{c} h_{t}=\underset{\tau }{\sum }Dropout\left(\alpha_{t\tau }\right)W_{v}x_{\tau }, \end{array}$$

where **W**_*v*_ ∈ *R*^*d*_*v*_.*dx*^ is used to transform **x**_*t*_ to the value space. It is possible to improve the self-attention performance by running *i* self-attention blocks (i.e., multihead attention) in parallel. This means that the key, query, and value matrices are split into a number of heads and projected. The individual splits are then passed into a self-attention block as described above. A multihead version of the equation (2) [32].

(3) $$\begin{array}{c} h_{t}=W_{o}\left(\begin{array}{c} \ldots \\ \underset{\tau }{\sum }Dropout\left(\alpha_{t\tau }^{i}\right)W_{v}^{i}x_{\tau }\\ \ldots \end{array}\right), \end{array}$$

where **W**_*o*_ ∈ *R*^*d*_*x*_.*ndv*^ is a projection matrix, *n* is the number of heads, and *i* is the head index.

### 3.2. Self-Attention Networks Complexity

The complexity of self-attention networks per layer is *O*(*n*^2^*d*), where *n* is the input sequence length and *d* is the embedding dimension. The self-attention networks compute the attention weights for each token with respect to every other token. Hence, it is *O*(*n*) operations for each token and therefore *O*(*n*^2^) for all the tokens. Moreover, the complexity of the number of sequential steps is *O*(1) where all *n* operations run in a single step (i.e., all the *n* tokens are processed in parallel).

On the other hand, the complexity per layer is *O*(*nd*^2^) for RNNs where the previous step's hidden states with the weight matrix multiplication run in *d*^2^ operations (i.e., *O*(*nd*^2^) for *n* steps). In addition, the complexity of the number of sequential steps is *O*(*n*) where all *n* operations run in *n* steps, respectively.

### 3.3. Architecture of the BERT Tagger

In addition to the multi-head attention sublayer, each transformer encoder layer has a fully connected feed-forward network (FFN). It consists of two linear transformations and a “GELU” nonlinear activation function in between. The FFN is applied to each word or token in the input sequence. Layer normalization and dropout are often used in the encoder layers.

**Figure 2:**
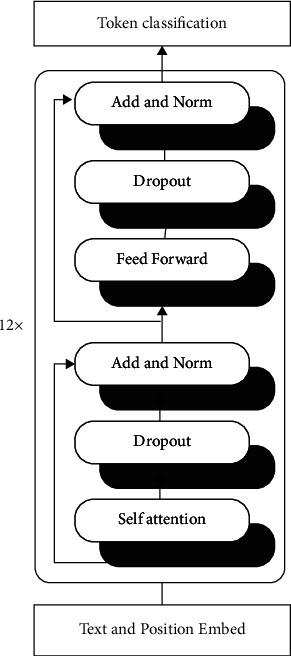
The BERT base tagger has 12 layers of transformer encoder layer and a token classification layer to predict the syntactic diacritics tags.

The BERT base tagger consists of stacking 12 layers of transformer encoder layers and a token classification layer to predict the syntactic diacritics tags. It has a hidden size of 768, 12 heads, and 110 M parameters. This token sequence classification setup is very close to the named-entity recognition (NER) task widely implemented using the BERT tagger [31].  Figure 2 shows the architecture of the BERT base tagger.

The English version of the BERT model was pretrained using the plain unlabeled text of the English Wikipedia and the Brown corpus. The objective function to train these models is based on masked language modeling (MLM), which is a self-supervised pretraining objective. It involves masking part of the input tokens and then training the BERT model to predict the masked tokens. The prediction task is formulated as a multi-class problem. Next sentence prediction (NSP) is the second objective of the training process for the BERT model [31].

A pretrained BERT model can be further optimized for the downstream tasks such as the syntactic diacritics predictions by adding an output layer. Then, small annotated datasets can be used to fine-tune this model and achieve high results for the downstream tasks. This technique is known as “transfer learning.”

The BERT model was originally designed for the English language. To solve the Arabic syntactic diacritics restoration task, we used a version of the BERT model pretrained for the Arabic language known as AraBERT [33]. It was chosen based on its empirical performance as detailed in Section V. We used two steps of fine-tuned models to achieve state-of-the-art results for the syntactic diacritics restoration. This novel approach is similar to the transfer and adapt (TANDA) method used for the answer sentence selection task in the question-answering domain [34].

The BERT model uses a word piece tokenizer [35] which divides the words into subwords. On the other hand, the syntactic diacritics restoration predicts a tag for each word in the input sequence. Similar to the BERT-based NER, the tag of the first token of each word is chosen as the final tag for each word during the prediction process.

In the next section, we will describe the datasets used for the syntactic diacritics restoration, a two-step fine-tuning process.

## 4. Data

We used a two-step fine-tuning approach to get the final results. This novel approach takes advantage of the available datasets for the syntactic diacritics restoration downstream task.

Our results for the syntactic diacritics restoration downstream task based on the BERT tagger are reported using the Penn Modern Standard Arabic (MSA) Arabic treebank (ATB) corpus [9]. The ATB catalog number LDC2005T20 (Part 3, version 2) of the linguistic data consortium (LDC) is used. From the Al-Nahar News text, part 3 dataset has 600 stories (340,281 words). A standard test dataset has 91 articles (approximately 52,000 words) that were published in 2002 between the fifteenth of October and the fifteenth of December, and is used for evaluation. This test set is used by other systems to report the state-of-the-art performance over years.

The ATB has annotations for syntactic (case-ending) and morphological diacritization, part-of-speech (POS) tagging, morphological segmentation, and syntactic parsing trees. The possible ATB syntactic diacritic tags are listed in Table 1.

An open-source corpus known as the Tashkeela corpus [25] is used for the two-steps fine-tuning setup. Using automatic web crawling techniques, this Classical Arabic (CA) corpus is collected from diacritized Islamic religious heritage books. The words of the corpus have morphological and syntactic diacritics. However, it does not have annotations for the location of the syntactic diacritics in the words.

We used some heuristics to guess the location of the syntactic diacritic mark for each word in the Tashkeela corpus. Hence, the final syntactic diacritic annotations are noisy and may be helpful for fine-tuning. We used 20,000,000 sentences from it to run the experiments.

In the two steps of fine-tuning, we used the Tashkeela corpus to fine-tune the BERT tagger. Consequently, we used the output model of this process for the second step of fine-tuning using the ATB corpus.

## 5. Experiments

All of our experiments were implemented using the Python simple transformers library [36] for BERT model fine-tuning and weights & biases platform [37] for tracking hyperparameters optimization. In addition, we ran all experiments on Google Colab Pro+ with a Tesla (R) V100 GPU, and the available memory was 54 GB.

### 5.1. Arabic BERT Models

There are a variety of Arabic BERT models that differ in some aspects such as model training, training dataset, and size of the model parameters.

AraBERT is a pretrained BERT utilized for the Arabic language. It is based on the BERT model and has the same architecture. In addition, it was built using a huge data from the Arabic Wikipedia and other resources. AraBERT was tested over many natural language processing (NLP) tasks and achieved a comparable result [33].

Arabic-BERT combines the architecture of the BERT model with CNNs which performs better than the BERT architecture only. Arabic Wikipedia, the Arabic copy of the open super-large crawled aggregated (OSCAR) corpus and other sources were used for model training over modern standard and dialectical Arabic [38].

Multi-Dialect-Arabic-BERT weights were initialized using AraBERT. Then, a ten million Arabic tweets corpus was used to fine-tune the model. In addition, ensemble techniques were used to enhance the model's performance [39].

The BERT multilingual base model (cased), is trained on more than one hundred languages which includes Arabic using the largest Wikipedia [31].

ARBERT and MARBERT were introduced in [40]. ARBERT was built for MSA. This model was trained over a group of datasets that have a huge amount of text (i.e., 6.2 billion tokens). On the other hand, MARBERT focused on MSA and dialectal Arabic (DA). A dataset with 1 billion Arabic tweets was used to train this model. Any tweet that has three or more Arabic words used in the training is eligible even if it has non-Arabic words.

The size of the dataset used in training and the size of parameters for each model are summarized in Table 2.

### 5.2. Results and Discussion

We start with an experiment to explore the previously mentioned Arabic BERT models. An ATB corpus was used in this experiment as described in Section 4. The official training set was divided into a training set (90%) and a development set (10%). The development set is used to evaluate the accuracy of the model during the training phase. The accuracy of the model is measured as the percentage of correct predictions of the Arabic syntactic diacritics (i.e., the last character of the stem of each word). We evaluated the trained models using the complement of the accuracy which is called the case-ending error rate (CEER) [3].

The number of training epochs was fifty in this experiment. In addition, the learning rate and the warmup steps were adjusted to their default values 4*e* − 5 and 0, respectively.

The AraBERT-v02 base achieved the best CEER over the test dataset of the ATB corpus, as shown in Table 3, because this model was trained using many Arabic resources [33]. We adopted this model as a reference model in the rest of the experiments.

To evaluate the model created using two steps of fine-tuning, the Tashkeela corpus was used in the first step to fine-tune the base model of AraBERT-v02 [33]. This model was built using 10 epochs, 2*e* − 5 learning rate, 0 value for warmup steps, 128 for sequence length to avoid out-of-memory issues, and default values for all of the other parameters.

For the second step, the ATB corpus was used to fine-tune the previous model. Fifty epochs were tried in addition to hyperparameters optimization for learning rate and warmup steps. A range between 1*e* − 5 and 4*e* − 4 was tried for the learning rate parameter, while 0, 500, 1000, and 1500 values were tried for warmup steps parameter. For all of the other parameters, we used the default values.

After finishing the hyperparameters optimization using the development set of the ATB corpus, we used the values of learning rate and warmup steps that achieved the best CEER to calculate the CEER of the model over the test dataset of the ATB corpus.

Table 4 illustrates the CEER of the one-step and two-step fine-tuning models over the test dataset of the ATB corpus. Two steps fine-tuning model outperforms one step with 0.34% CEER.

The two-steps fine-tuning approach which is similar to the Transfer and Adapt (TANDA) [34], achieved better results since it first fine-tunes the model using a large dataset (i.e., the transfer step) and then fine-tunes the model again using a domain-specific dataset (i.e., the adaptation step).

Finally, a comparison between the proposed system and the state-of-the-art systems over the test dataset of ATB corpus is introduced in Table 5. These systems measure the performance using two metrics. The first metric is “All WER” which counts all diacritization errors. “Morph WER” is the second metric that ignores the last character diacritization errors for each word. Hence, the “CEER” for these systems equals the difference between the previous two metrics. Our proposed approach (i.e., BERT tagger) achieved the best result with 2.94% CEER.

## 6. Conclusion

The Arabic syntactic diacritic restoration is often handled with taggers based on LSTM networks. In this paper, we presented a BERT tagger to predict the syntactic diacritics. The tagger is based on self-attention networks which run in parallel and model the long-term dependencies better than LSTM taggers. On the standard Arabic treebank corpus, our BERT approach reports a new state-of-the-art CEER. This result was achieved without using handcrafted features like those used in the LSTM taggers. For future work, we aim to combine the hidden Markov models (HMMs) approach used to predict the morphological diacritics with our BERT tagger to predict syntactic diacritics. This may lead to the best results for restoring the full word's Arabic diacritics.

## Figures and Tables

**Table 1:** The ATB dataset syntactic diacritics tags (marks) definitions.

ATB tag	Meaning	Ex.	Pron.
B-NCE	Diacritic tag is not given or Skoon	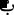	/b/

CASE-DEF-GEN	Kasrah	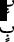	/b//i/
CASE-INDEF-GEN	Nunation of Kasrah		/b//in/

CASE-DEF-NOM	Dammah	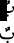	/b//u/
CASE-INDEF-NOM	Nunation of Dammah		/b//un/

CASE-DEF-ACC	Fathah	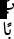	/b//a/
CASE-INDEF-ACC	Nunation of Fathah		/b//an/

NUM	Numbers	—	—

**Table 2:** Characteristics of the tested Arabic BERT models.

Model	Dataset size	Model parameters (M)
AraBERT-v02 base [33]	77 GB/8.6 B	135
Arabic BERT [38]	95 GB/8.2 B	110
Multi-dialect Arabic BERT [39]	—	110
BERT multilingual base model [31]	—	110
ARBERT [40]	61 GB/6.2 B	163
MARBERT [40]	128 GB/15.6 B	163

**Table 3:** CEER of the different Arabic BERT models on the ATB test dataset.

Model	CEER (%)
AraBERT-v02 base [33]	**3.36**
Arabic BERT [38]	11.26
Multi-dialect Arabic BERT [39]	12.38
BERT base multilingual cased [31]	6.07
ARBERT [40]	10.76
MARBERT [40]	12.77

**Table 4:** CEER of one-step and two-step fine-tuning model.

Model	CEER (%)
AraBERT-v02 base and one step fine-tuning using ATB dataset	3.28
AraBERT-v02 base and two steps fine-tuning using Tashkeela and ATB datasets	**2.94**

**Table 5:** The comparison between the proposed BERT model and the state-of-the-art systems on the ATB test dataset.

Method	All WER	Morph WER	CEER (%)
MaxEnt tagger [10]	18.00%	7.90%	10.10
Rule-based tagger [41]	—	—	9.97
MADA tagger [42]	14.90%	5.50%	9.40
Random forest tagger [28]	13.70%	4.30%	9.40
Scoring of a language model [5]	12.50%	3.10%	9.11
Confused subset resolution [43]	11.60%	3.00%	8.60
Scoring of a language model [16]	10.87%	3.00%	7.87
SVM tagger [29]	—	—	6.8
MADAMIRA + character RNN tagger [21]	8.40%	2.30%	6.10
Character RNN tagger [20]	9.07%	4.34%	4.73
Word level MaxEnt/BiLSTM tagger [29]	—	—	5.3
Word level MaxEnt/BiLSTM tagger + distillation of knowledge + embeddings based on characters [4]	—	—	4.3
BERT tagger (two steps fine-tuning)	—	—	2.94

## Data Availability

The Arabic TreeBank (reference [8] in our text) catalog number LDC2005T20 (Part 3, version 2) (https://catalog.ldc.upenn.edu/LDC2005T20) data used to support the findings of this study is not free and can be ordered from Linguistic Data Consortium (LDC). On the other hand, the open-source Tashkeela corpus is used to support this study and it is available at https://www.kaggle.com/datasets/linuxscout/tashkeela. It is cited at relevant places within the text as reference [25].
